# HERV-K Modulates the Immune Response in ALS Patients

**DOI:** 10.3390/microorganisms9081784

**Published:** 2021-08-23

**Authors:** Giannina Arru, Grazia Galleri, Giovanni A. Deiana, Ignazio R. Zarbo, Elia Sechi, Marco Bo, Maria Piera L. Cadoni, Davide G. Corda, Claudia Frau, Elena R. Simula, Maria Antonietta Manca, Franca Galistu, Paolo Solla, Roberto Manetti, Gian Pietro Sechi, Leonardo A. Sechi

**Affiliations:** 1 Department of Clinical, Surgical and Experimental Sciences, University of Sassari, Viale San Pietro 8, 07100 Sassari, Italy; parentina@yahoo.com (G.A.); galleri@uniss.it (G.G.); gdeiana@uniss.it (G.A.D.); irzarbo@uniss.it (I.R.Z.); eliasechi@icloud.com (E.S.); mariapieracadoni@libero.it (M.P.L.C.); dvcorda@tiscali.it (D.G.C.); clafrau@hotmail.it (C.F.); psolla@uniss.it (P.S.); rmanetti@uniss.it (R.M.); 2 Department of Biomedical Sciences, University of Sassari, Viale San Pietro 43b, 07100 Sassari, Italy; 30039536@studenti.uniss.it (M.B.); simulaelena@gmail.com (E.R.S.); m.anto.manca@gmail.com (M.A.M.); 3 Laboratory of Clinical Pathology, AOU Sassari, Via Monte Grappa 43, 07100 Sassari, Italy; franca.galistu@aousassari.it; 4 SC Microbiologia, Azienda Ospedaliera Universitaria, 07100 Sassari, Italy; 5 Mediterreanean Center for Diseases Control, 07100 Sassari, Italy

**Keywords:** amyotrophic lateral sclerosis, HERV-K, antigenic peptides, humoral and cell mediated immune response

## Abstract

Human endogenous retrovirus (HERV)-K env-su glycoprotein has been documented in amyotrophic lateral sclerosis (ALS), where HERV-K env-su _19–37_ antibody levels significantly correlated with clinical measures of disease severity. Herein, we investigated further the humoral and cell-mediated immune response against specific antigenic peptides derived from HERV-K in ALS. HERV-K env glycoprotein expression on peripheral blood mononuclear cells (PBMCs) membrane and cytokines and chemokines after stimulation with HERV-K env _19–37_ and HERV-K env _109–126_ were quantified in patients and healthy controls (HCs). HERV-K env glycoprotein was more expressed in B cells and NK cells of ALS patients compared to HCs, whereas HERV-K env transcripts were similar in ALS and HCs. In ALS patients, specific stimulation with HERV-K env _109–126_ peptide showed a higher expression of IL-6 by CD19/B cells. Both peptides, however, were able to induce a great production of IFN-γ by stimulation CD19/B cells, and yielded a higher expression of MIP-1α and a lower expression of MCP-1. HERV-K env _19–37_ peptide induced a great production of TNF-α in CD8/T cells. In conclusion, we observed the ability of HERV-K to modulate the immune system, generating mediators mainly involved in proinflammatory response.

## 1. Introduction

Amyotrophic lateral sclerosis (ALS) is a neurodegenerative disease that affects both upper and lower motor neurons, determining progressive paralysis and death, within 3–5 years post diagnosis. About 5–10% of ALS cases are familial, the remaining are apparently sporadic of unknown etiology [1]. A poor knowledge of ALS pathophysiology is the major reason for the absence of specific diagnostic tests in clinical practice and the lack of an effective treatment. Thus, it is important to find reliable diagnostic and prognostic markers that can guide the clinical diagnostic process at an early stage of the disease. There is no cure for ALS, and the FDA-approved drugs are only marginally effective in slowing the progression of the disease by a few months [1]. Advances in understanding pathogenic mechanisms are essential to identify new possible therapeutic interventions. Several studies have demonstrated the presence of reverse transcriptase activity in serum samples of both ALS patients and their unaffected relatives [2,3], indicating that a common inherited endogenous retrovirus may be the trigger of the disease [4]. Endogenous retroviral sequences constitute 8% of human genomes, in which they have been integrated through repeated infections during evolution [5]. In particular, RNA sequences of the human endogenous retrovirus of the K family (HERV-K) have been detected in motor neurons of ALS patients [6,7], and it has been observed that increased expression of HERV-K envelope protein in upper and lower motor neurons was neurotoxic and able to cause cellular degeneration [7], thus indicating the reactivation of HERV-K in the affected tissues. Moreover, in a transgenic mouse model, increased HERV-K envelope protein expression in motor neurons induces a clinical and pathological phenotype that resembles ALS, strongly suggesting that HERV-K may play a contributing role in the pathophysiology of this disorder [7].

Recently, we investigated the specific humoral immune response against four antigenic peptides derived from the HERV-K env-su glycoprotein in serum and cerebrospinal fluid (CSF) of ALS patients, reporting a significant immune response directed toward the HERV-K env-su _19–37_ peptide both in serum and CSF of ALS patients, but not in healthy controls (HCs). In addition, we observed a specific intrathecal IgG synthesis against HERV-K peptides and a functionally intact blood–brain barrier in most of the patients analyzed, indicating that there is active antibody production within the CNS. Moreover, in ALS patients, the HERV-K env-su _19–37_ antibody levels were significantly correlated with clinical measures of disease severity, both in serum and CSF [8]. In this paper, we shed light on the role of HERV-K in the pathophysiology of ALS by evaluating the humoral and cell-medi ated immune response to HERV-K in ALS patients compared to HCs, using different methodological approaches: (1) cytometric analysis to quantify HERV-K env-su glycoprotein expression on peripheral blood mononuclear cells (PBMCs) membrane and test the T and B mediated immune response after antigenic stimulation with HERV-K env _19–37_ and HERV-K env _109–126_, by quantification of Interleukin-6 (IL-6), Interferon- γ (IFN-γ), Tumor Necrosis Factor-α (TNF-α), Macrophage Inflammatory Protein-1α (MIP-1α), and Monocyte Chemoattractant Protein-1 (MCP-1); (2) investigation of HERV-K transcripts in PBMCs by RT qPCR method; (3) analysis by ELISA of specific antibodies against env-surface peptides; and (4) quantification of IgG1 and IgG4 subclasses by nephelometry. We detected IgG1 plasma levels significantly higher in ALS patients compared to HCs; conversely, no difference between the two groups was observed for IgG4 plasma levels.

The results obtained highlighted the ability of HERV-K to modulate the immune system at different levels, generating mediators mainly involved in proinflammatory response. Of note, despite depletion, B cells of ALS patients express higher levels of HERV-K env protein compared to HCs feeding the inflammatory loop, by induction of a continuous proinflammatory stimulus of T lymphocytes sustained by exposition of viral protein on B lymphocytes membrane.

## 2. Material and Methods

The study protocol was approved by the institutional review board of the Sassari University Hospital (Azienda Ospedaliero-Universitaria, Sassari, Italy: IRB number 2395/2016). All participants provided written informed consent.

### 2.1. Patients

For this study, we consecutively included, from January 2016 to December 2019, (1) sporadic, newly diagnosed ALS (sALS) patients, seen at the Neurology Unit of the University Hospital of Sassari, (2) long-survivor ALS patients reported by primary doctors and doctors in the Sassari local district, and (3) age and sex matched HC subjects from the Blood Transfusion Centre of Sassari. The diagnosis of ALS was made by the treating neurologists, according to the revised El Escorial criteria [9]. Clinical measures of ALS severity were evaluated through the ALS Functional Rating Scale—Revised [10]. Table 1 shows the demographic data of investigated groups and the different methodological approaches used. Patients with known infections at the time of study enrollment were excluded.

### 2.2. Blood Samples

All the subjects recruited for the study provided 20 mL of blood in EDTA tubes. PBMCs were isolated by centrifugation on a discontinuous density gradient (Ficoll); the obtained cells were washed twice in phosphate-buffered saline (PBS) and counted. They were then divided into two aliquots, one of which was frozen in dimethylsulfoxide solution and stored in liquid nitrogen to maintain cell viability in view of the flow cytometry analysis. The second aliquot was used for RNA extraction and quantification of HERV-K env transcripts by RT-qPCR (see below).

### 2.3. Antigens

Synthetic peptides derived from HERV-K env protein were used in this study for the antigenic stimulation. Peptides, described in detail in the previous paper [8], were synthesized commercially at 90% purity (LifeTein, South Plainfield, NJ 07080, USA).

### 2.4. Antibody Reactivity against HERV-K Immunogenic Peptides

Antibody response against two antigenic peptides derived from HERV-K envelope proteins, namely HERV-K env _19–37_ [VWVPGPTDDRCPAKPEEEG] and HERV-K env _109–126_ [RPKGKTCPKEIPKGSKNT], belonging to the surface region of the protein, was assessed with a previously described methodology [8]. Briefly, 96-well plates (NuncImmuno™ Plates, Maxi Sorp, Nalgen Nunc International, Sigma Aldrich, St. Louis, USA) were coated overnight at 4 °C with 10 µg/mL of each peptide in a solution 0.05 M of carbonate-bicarbonate, pH 9.5 (Sigma Aldrich, St. Louis, MO, USA). Plates were washed twice with 0.1% Tween-20 (in TBS) and blocked with 5% skimmed dried milk in PBS for 1 h at room temperature (25 °C). Plasma samples (1:1000 dilution) were added and incubated for 2 h at room temperature. Secondary antibody was alkaline phosphatase-conjugated goat anti-human immunoglobulin G polyclonal Ab (1:1000; Sigma Aldrich, St. Louis, MO, USA). Plates were washed between each incubation. Alkaline phosphatase was detected with para nitrophenyl phosphate (Sigma). Absorbance was read at 405 nm on a plate reader (SpectraMax Plus 384, Molecular Devices, Sunnyvale, CA, USA). All incubation volumes were 100 µL/well. Each sample was run in duplicate, and normalization was performed with a positive control (absorbance reactivity set at 1.0 arbitrary units) included in each assay. Background activity was calculated as the mean signal of an immobilized peptide with secondary Ab alone.

In our previous ELISA study, these two peptides only triggered a significantly increased antibody response in patients with ALS, the HERV-K env _109–126_, only in serum, the HERV-Kenv _19–37_ in serum, and CSF [8].

### 2.5. Cell Preparation and Antigenic Stimulation

Cells of ALS patients and HCs were quickly thawed using RPMI, added to 10% of autologous human serum, and counted. They were then divided into 4 tubes, each containing 1,000,000 cells. The conditions were: positive and negative controls and the two HERV-K env stimulation peptides.

Positive control included: 500 µL of RPMI, 500 µL of cells suspension, the PMA (phorbol 12-myristate 13-acetate) 50 µg/mL, and calcium ionophore A23187 0.5 µg/mL (Sigma Al drich). Negative control included: 500 µL of RPMI and 500 µL of cells suspension. Antigenic stimulation sample were performed with: 500 µL of RPMI, 500 µL of cells suspension, the costimulatory mAB CD28/49d d 5 µL/mL, and two different peptides (HERV-K env _19–37_ and HERV-K env _109–126_, 20 µM). The culture tubes were incubated in a diagonal position at 37 °C of 5% CO_2_ for 8 h; the first 2 h of incubation were without Brefeldin A (BFA) to enable antigen processing by antigen presenting cells (APC). The BFA (10 µg/mL) was included for the final 6 h of activation in order to inhibit the secretion and paralysis of the Golgi apparatus. Afterwards, the samples were centrifuged for 10 min at 1200 rpm, then the supernatant was aspired and discerned. The pellet was vortexed and the samples were incubated for 15 min in the dark with 500 µL of 1 × Fixation Buffer (R&D) at room temperature (RT), than supplemented with 500 µL of Stain Buffer (BD Pharmigen, San Diego, CA USA). Cells were centrifuged for 10 min at 1200 rpm, and then the supernatant was discarded by inversion. The samples were gently resuspended in 2 mL of Stain Buffer (BD Pharmigen) and incubated at 4 °C overnight (o/n).

### 2.6. Intracellular Cytokine Staining and Phenotyping Analysis

The following day, cells were centrifuged for 10 min at 1200 rpm, the supernatant was discarded by inversion, then 200 µL of permeabilizing solution (0.05% saponin in PBS solution) were added to each sample and incubated for 10 min. Subsequently, the solution containing 100 µL of cells suspension and 100 µL of permeabilizing solution was divided into two tubes. Mix of antibodies was added for marking and samples were further incubated for 30 min in the dark. After washing with 2 mL of permeabilizing solution, centrifuging for 10 min at 1200 rpm, and discarding the supernatant by inversion, cells were resuspended in 300 µL of FACS Flow solution. The labeled cells were analyzed by flow cytometry on FACSCanto™ using FACSDiva 2.2 (Becton and Dickinson, Milano, Italia).

### 2.7. Cytokines and Phenotyping Antibodies

By using the intracellular cytokine (ICC) method, we evaluated in PBMCs, the immune response T and B mediated after stimulation with specific peptides. The ICC method permits to quantify the intracellular production of cytokines in specific subpopulations of CD4, CD8, CD19, CD14, and CD56/16 following specific peptide stimulus. In particular, monoclonal antibodies (mAbs) CD3 FITC, CD 19 PE, CD8 ACP-Cy7, INF-γ PE-Cy7, and IL-6 APC were used for the analysis of T and B lymphocytes, while monocytes, natural killer (NK) cells and T lymphocytes were detected using: CD3 FITC, CD14 APC-Cy7, MIP1-α PE, TNF -α PE Cy 7, CD56/CD16 PerCP-Cy5.5, MCP-1 APC, and CD8 APC-Cy7. The antibodies were used at the manufacturer’s recommended concentrations (BD Biosciences, San Diego, CA USA).

### 2.8. HERV-K env Protein Detection

The performance characteristics for the flow cytometry to detect HERV-K env protein on the plasma membrane were as follows: anti-HERV-K env mouse monoclonal antibody(clone 215/H4) (Space Import) and secondary allophycocyanin crosslinked goat antimouse IgG (Invitrogen, Thermo Fisher Scientific, CA USA) were used. The isotype control was a preimmune rabbit serum (Santa Cruz Biotechnology, Inc, Heidelberg, Germany). To determine the phenotype of cell subpopulations, PBMCs were stained with monoclonal antibodies (mAbs) CD3 FITC, CD19 PE, CD8 ACP, CD56/CD16 PerCP-Cy5.5, and CD14 APC-Cy7 (BD Biosciences, San Diego CA). For each sample, 1 × 106 cells in a 100 µL final volume were incubated in the dark for 30 minutes on ice. Stained cells were analyzed by flow cytometry on FACSCanto™ using FACSDiva 2.2 (Becton and Dickinson), a total of at least 5 × 104 events for each sample were acquired.

### 2.9. RNA Extraction and Real-Time RT-PCR

RNAs were extracted from 3 × 106 PBMCs isolated from peripheral blood by RNeasy^^®^^ Plus Mini Kit (Qiagen, Hilden, Germany) and treated with RNase free DNase (Qiagen). The concentration and the quality of RNA were evaluated with the Thermo Scientific ™ NanoDrop 2000 spectrophotometer (Thermo Fisher Scientific). Reverse transcription of 1 µg of total RNA into cDNA was performed using SuperScript™ III First-Strand Synthesis SuperMix (Invitrogen) according to manufacturer’s instructions. Gene expression levels for HERV-K env and glyceraldehyde 3-phosphotate dehydrogenase (GAPDH) were determined by quantitative PCR performed on C1000 Touch Thermal Cycler (Bio Rad CFX96 Real-Time System, Hercules CA, USA). PCR primers and probes for quantitative PCR were designed by the Beacon Designer software (PREMIER Biosoft International Corina Way Palo Alto, CA, USA), which designs the most favorable primers and probes for a specific target region, and are listed in Table 2.

Absence of DNA contamination in the RNA samples was confirmed when target gene was not detected by PCR when RT was omitted during reverse transcription. The amount of RNA in PBMCs was expressed as relative levels to controls samples after normalization with the glyceraldehyde-3-phosphate dehydrogenase (GAPDH) invariant housekeeping gene. Data were expressed according to the 2^−ΔCt^ method.

### 2.10. IgG 1 and IgG 4 Subclasses Analysis

Forty-eight ALS serum samples, newly diagnosed patients (*n* = 27), long-survivor patients (*n* = 21), were analyzed for the quantitative determination of IgG1 (N AS IgG1 (Siemens, PA, USA) ref: OQXI09) and IgG4 (N Latex IgG4 (Siemens) ref: OPAU03) subclasses using immuno-nephelometry on the BN II System. Fifty-eight, HCs serum samples, matched by age and sex were also analyzed. The assay protocols for IgG in serum and CSF, respectively, were given in the BN II System Instruction Manual and software of the instrument. Screening at the starting dilution of 1:2000 in serum to arrive to a concentrate solution of the sample until 1:1 and titration of the positive cases were performed.

### 2.11. Statistical Analysis

All data obtained were analyzed using GraphPad Prism 8.4.1 software (GraphPad Software, San Diego, CA, USA). Continuous variables were presented as the mean ± standard deviation (SD), and categorical variables were presented as numbers and percentages. The analysis between two groups was executed using the non-parametric Mann–Whitney test and a value of *p* < 0.05 was considered statistically significant. The diagnostic value of the indirect ELISA assays was evaluated by the receiver operating characteristic (ROC) curve. The optimal cut off values were chosen according to ROC analysis, setting the most appropriate specificity and sensitivity for all serum and CSF samples measured. In ALS patients, linear regression for the different serum HERV-K antibodies levels against HERV-K env expression on the B cells membrane, was calculated using Spearman test for non-parametric data.

## 3. Results

### 3.1. ELISA

**Figure 1:**
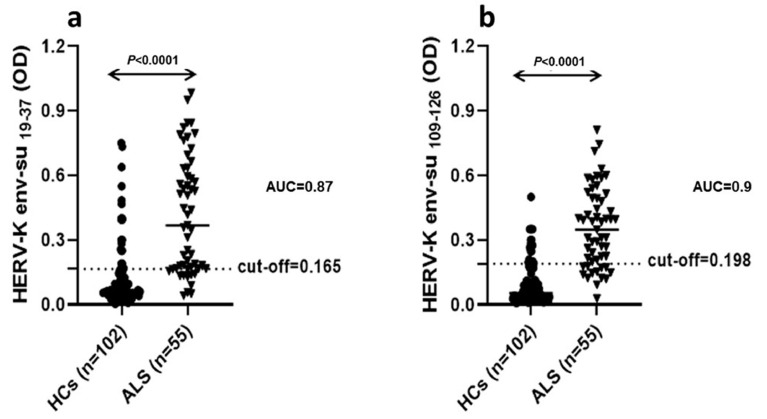
Indirect ELISA showing the antibody OD against the HERV- env-su _19–37_ peptide (**a**) and the HERV-K env su _109–126_ (**b**). A total of 55 samples from ALS patients and 102 HCs were screened for their reactivity against plate-coated with HERV-K env _19–37_ and HERV-K env _109–126_ peptides in serum. Antibodies against both peptides are statistically higher in the sera of ALS patients in comparison to HCs. Scatter plots present median values with interquartile range. Area under ROC curve (AUC), as well as *p* value, are displayed. Cut-off values for positivity, calculated by ROC analysis are indicated by dashed lines.

A total of 55 serum samples from ALS patients and 102 serum samples from HCs were analyzed. Antibodies (Abs) against HERV-K env-su _19–37_ were found in the sera of 78.2% ALS patients, whereas only 19.6% of HCs were positive (AUC = 0.87, *p* < 0.0001) (Figure 1a). Regarding HERV-K env-su _109–126_ peptide, 76% of ALS patients and 16% of HCs were positive for Abs in sera (AUC = 0.9, *p* < 0.0001) (Figure 1b).

### 3.2. Distribution of Blood Cells Subset

The distribution of the PBMCs subset in HCs and ALS patients is shown in Table 3. The percentage of CD3- was lower in ALS patients compared to HCs (*p* = 0.015), as was the percentage of B lymphocytes CD3-/CD19+ (*p* = 0.034). Vice versa, there was a statistically significant increase in the total lymphocyte percentage of patients with ALS vs. HCs (*p* = 0.02) although, in the specific subpopulations, both CD4/T-helper and CD8/cytotoxic showed no significant differences. Likewise, no statistically significant difference in the percentage of CD14/monocytes and CD56/16/NK cells was observed between the two groups.

### 3.3. Expression of HERV-K env Protein on PBMCs

**Figure 2:**
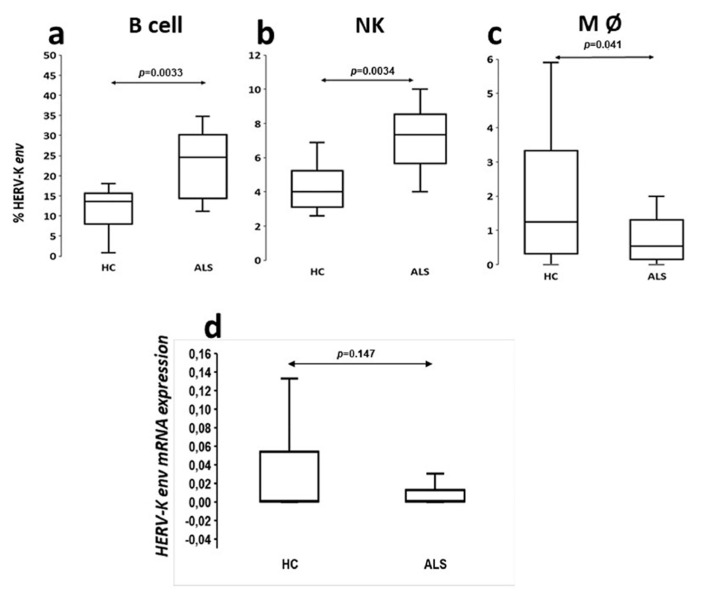
Expression of HERV-K env protein and levels of HERV-K env mRNAs in PBMC of ALS patients and controls. The protein expression was evaluated by Flow cytometry of HERV-K env protein in PBMC subpopulations of ALS patients and HCs; in particular: (**a**) in B cells CD19; (**b**) in Natural killer cells CD56/16; (**c**) in monocytes CD14; (**d**) Detection of HERV-K env mRNAs in PBMCs of ALS patients and HCs, by real-time RT-PCR (means of three experiments run in duplicate, calculated by the 2^−ΔCt^ methods). RT-PCR: reverse transcription-polymerase chain reaction. PBMC: peripheral blood mononuclear cell; and ALS: amyotrophic lateral sclerosis.

The analysis of retroviral expression on lymphomonocytes showed that HERV-K env protein was expressed with greater frequency in patients’ B cells (ALS compared to HCs, mean ± SD = 27.6 ± 7.97 vs. 13.7 ± 5.97, *p* = 0.0033), as shown in Figure 2a, and NK cells (ALS vs. HCs, mean ± SD = 7.4 ± 1.96 vs. 4 ± 1.44, *p* = 0.0034) (Figure 2b). Conversely, the env protein of HERV K was significantly present on the monocyte membrane of HCs compared to ALS patients (ALS vs. HCs, mean ± SD = 0.67 ± 0.62 vs. 1.97 ± 1.93, *p* = 0.041) (Figure 2c). Furthermore, HERV-K env protein appeared to be poorly expressed on the CD4+ and CD8+ lymphocyte membranes of both patients and controls (data not shown).

### 3.4. Levels of HERV-K env mRNAs in PBMC of ALS Patients and Controls

HERV-K *env* gene expression was quantified by real-time PCR in PBMCs from ALS patients (*n* = 36) and HCs (*n* = 44) collected over the study time. The expression of HERV-K *env* mRNA in sALS patients (mean ± SD = 0.0011 ± 0.032) was not significantly different from that observed in HCs (mean ± SD = 0.00157 ± 0.053; *p* = 0.231) (Figure 2d).

### 3.5. Frequency of Expression of Cytokines and Chemokines Following Specific Antigen Stimulus with Retroviral Peptides B Cells Activation

**Figure 3:**
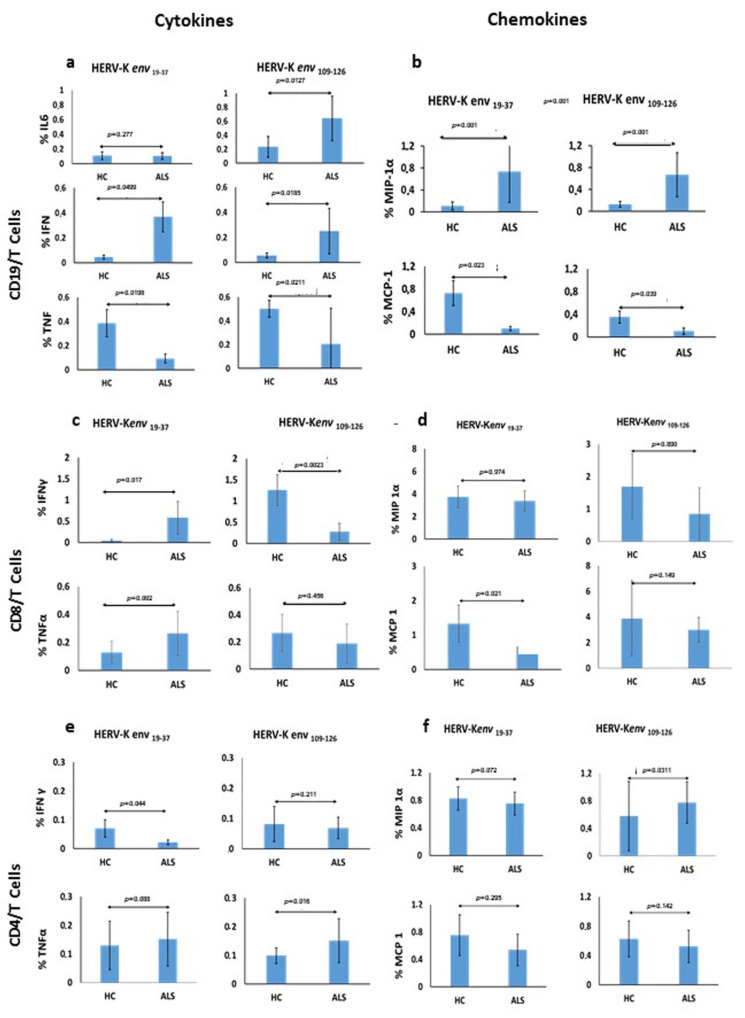
Frequencies of CD19/B cells, CD8/T cells and CD4/T cells producing cytokines and chemokines analyzed by Flow cytometry assay. (**a**) Percentage of CD19/B cells secreting IL-6, IFN-γ, and TNF-α after 8 h incubation with HERV-K env _19–37_ (on the left) and HERV-K env _109–126_ (on the right); (**b**) Percentage of CD19/B cells secreting MIP-1 α and MCP-1 after 8 h incubation with HERV-K env _19–37_ (on the left) and HERV-K env _109–126_ (on the right); (**c**) Percentage of CD8/T cells secreting IFN-γ and TNF-α after 8 h incubation with HERV-K env _19–37_ (on the left) and HERV-K env _109–126_ (on the right); (**d**) Percentage of CD8/T cells secreting MIP-1 α and MCP-1 after 8 h incubation with HERV-K env _19–37_ (on the left) and HERV-K env _109–126_ (on the right); (**e**) Percentage of CD4/T cells secreting IFN-γ and TNF-α after 8 h incubation with HERV-K env _19–37_ (on the left) and HERV-K env _109–126_ (on the right); (**f**) Percentage of CD4/T cells secreting MIP-1 α and MCP-1 after 8 h incubation with HERV-K env _19–37_ (on the left) and HERV-K env _109–126_ (on the right).

PBMCs activation against the (HERV-K env _19–37_ and HERV-K env _109–126_) peptides in 14 ALS patients and 11 HCs blood samples was assessed by intracellular cytokine flow cytometry. Patients’ response was compared to that of HCs by analyzing the percentage of cells secreting IL-6, IFN-γ, TNF-α, MIP-1 α, and MCP-1 in B cells. A significant difference in the expression of IL-6 by B cells was observed after stimulation with HERV-K env _109–126_ peptide in ALS patients compared to HCs with statistically significant *p* value (ALS vs. HCs mean ± SD = 0.641 ± 0.31 vs. 0.234 ± 0.14; *p* = 0.0127) (Figure 3a). Vice versa, no difference in IL-6 expression was observed after stimulation with HERV-K env _19–37_ peptide (Figure 3a). Regarding the activation of B cells producing IFN-γ, ALS patients showed a significant increase in percentage of IFN-γ compared to HCs after stimulation with both HERV-K env _19–37_ (ALS vs. HCs, mean ± SD = 0.369 ± 0.121 vs. 0.044 ± 0.016; *p* = 0.049) (Figure 3a) and HERV-K env _109–126_ (ALS vs. HCs, mean ± SD = 0.250 ± 0.18 vs. 0.055 ± 0.02; *p* = 0.0185) (Figure 3a). Conversely, TNF-α was strongly expressed in HCs compared to ALS patients B cells, with a statistically significant difference for both peptides (ALS vs. HCs, mean ± SD = 0.0927 ± 0.038 vs. 0.386 ± 0.112; *p* = 0.0198) (ALS vs. HCs, mean ± SD = 0.206 ± 0.29 vs. 0.501 ± 0.06; *p* = 0.021), respectively (Figure 3a). PBMCs stimulated with the HERV-K env _19–37_ peptide showed a significant increase in percentage of MIP-1 α secreting B cells in ALS patients in respect to HCs (ALS vs. HCs mean ± SD = 0.733 ± 0.51 vs. 0.107 ± 0.07; *p* = 0.001) (Figure 3b). Similarly, we observed a significant difference in responses following HERV-K env _109–126_ incubation regarding MIP-1 α expression among ALS patients and controls (ALS vs. HCs, mean ± SD = 0.669 ± 0.42 vs. 0.130 ± 0.05 *p* = 0.001) (Figure 3b). However, PBMCs activation against the selected HERV-K env peptides displayed a greater expression of the chemokine MCP-1 in HCs compared to ALS patients with a significant difference, both for HERV-K env _19–37_ (ALS vs. HCs, mean ± SD = 0.102 ± 0.038 vs. 0.726 ± 0.21; *p* = 0.023) and HERV-K env _109–126_ (ALS vs. HCs, mean ± SD = 0.107 ± 0.058 vs. 0.352 ± 0.11; *p* = 0.039) (Figure 3b).

### 3.6. CD8+ T Cells Stimulation

We also investigated the CD8+T cells response after 8h of incubation with specific HERV-K env peptides, analyzing the percentage of CD8+ cells secreting IFN-γ, TNF-α, MIP-1 α, and MCP-1.

A significant difference in the expression of IFN-γ by CD8+ T cells was observed after stimulation with HERV-K env _19–37_ peptide in ALS patients with respect to HCs with a statistically significant *p* value (ALS vs. HCs, mean ± SD = 0.582 ± 0.385 vs. 0.044 ± 0.022; *p* = 0.017) (Figure 3c). Conversely, a higher expression of IFN-γ was observed in the HC group after incubation with HERV-K env _109–126_ (ALS vs. HCs, mean ± SD = 0.277 ± 0.196 vs. 1.26 ± 0.365; *p* = 0.0023) (Figure 3c). The specific antigen stimulus with HERV-K env _19–37_ peptide produced significant differences in TNF-α expression between ALS patients and HCs (ALS vs. HCs, mean ± SD = 0.265 ± 0.158 vs. 0.127 ± 0.081; *p* = 0.002) (Figure 3c). We did not observe any statistically significant difference in the percentage of CD8+ T cells producing TNF-α after HERV-K env _109–126_ stimulus (Figure 3c). No difference was observed in MIP-1 α expression after incubation of PBMCs with both peptides (Figure 3d). In contrast, a higher expression of MCP-1 by HCs in comparison to ALS patients was detected after stimulation with HERV-K env _19–37_ peptide (ALS vs. HCs, mean ± SD = 0.673 ± 0.124 vs. 1.33 ± 0.539; *p* = 0.021) (Figure 3d) whereas no difference in MCP-1 expression was observed after HERV-K env _109–126_ stimulation (Figure 3d). This is still consistent with the above data where, in healthy subjects, we can observe increased expression of viral peptides on the membranes of B cells and monocytes. In fact, the MCP-1 has a chemotactic function for both monocytes and B cells.

### 3.7. CD4+ T Cells Stimulation

PBMCs activation against the selected HERV-K env peptides (HERV-K env _19–37_ and HERV-K env _109–126_) was assessed in ALS patients and HCs analyzing the percentage of CD4+ cells secreting IFN-γ, TNF-α, MIP-1 α, and MCP-1. We detected a significant difference in the expression of IFN-γ by CD4+ T cells in HCs compared to ALS patients after stimulation with HERV-K env _19–37_ peptide (ALS vs. HCs mean ± SD = 0.023 ± 0.082 vs. 0.07 ± 0.03; *p* = 0.044) (Figure 3e). Conversely, we did not observe any difference in the percentage of CD4+ T cells producing IFN-γ after HERV-K env _109–126_ stimulus (Figure 3e). In the same way, no difference was detected regarding TNF-α expression in CD4+ T cells incubated with HERV-K env _19–37_ peptide (Figure 3e). Conversely, a higher expression of TNF-α in ALS patients compared to HCs was found after stimulation with HERV-K env _109–126_ (ALS vs. HCs mean ± SD = 0.151 ± 0.076 vs. 0.0990 ± 0.027; *p* = 0.016) (Figure 3f). Next, we analyzed the frequency of CD4+ T cells producing MIP-1 α and MCP-1. No difference in MIP-1 α expression was observed after stimulation with HERV-K env _19–37_ peptide (Figure 3f), whereas a significant difference between ALS patients and HCs was detected after incubation with HERV-K env _109–126_ (ALS vs. HCs median ± SD = 0.776 ± 0.3 vs. 0.579 ± 0.43) (Figure 3f). In contrast, CD4+ T cells secreting MCP-1 did not show differences in response to stimulation with both peptides in ALS patients and HCs (Figure 3f).

### 3.8. IgG 1 and IgG 4 Subclasses Levels in the Sera of ALS Patients and HCs

**Figure 4:**
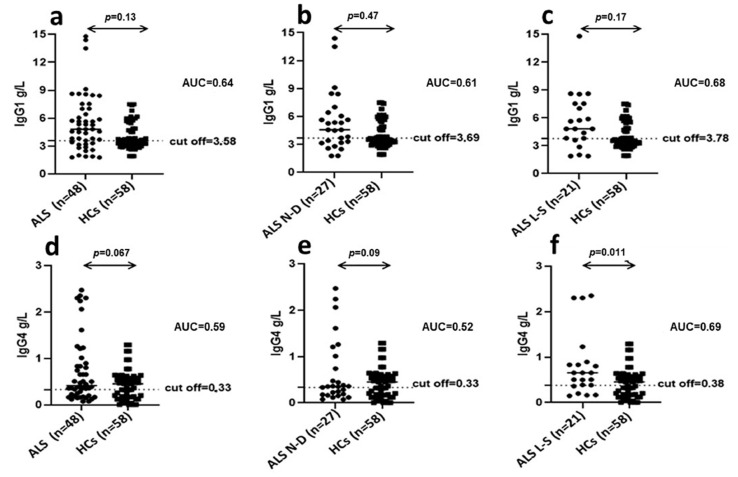
IgG 1 and IgG 4 subclasses levels in the sera of ALS patients and HCs. IgG1 and IgG4 levels in the total ALS group (*n* = 48) (**a** and **d**, respectively); in N-D (*n* = 27) (**b** and **e**, respectively) and, in L-S (*n* = 21) (**c** and **f**, respectively) in comparison to HCs group (*n* = 58) Scatter plots present mean values ± SD. Area under ROC curve (AUC) as well as *p* value are displayed in the top-right corner. Cut-off values for positivity, calculated by ROC analysis, are indicated by dashed lines.

A significant difference was observed comparing IgG1 levels in the total ALS group (*n* = 48) (newly diagnosed, N-D; *n* = 27 plus long-survivors, L-S; *n* = 21), in comparison to HCs group (*n* = 58) (ALS vs. HCs, mean ± SD = 4.82 ± 3.06 vs. 3.56 ± 1.43; *p* = 0.013). In fact, 73% total ALS patients showed increased IgG1 levels vs. only 47% of HCs (AUC = 0.64) (Figure 4a). Conversely, no difference was observed comparing IgG1 levels in the N-D ALS patients with respect to HCs (ALS vs. HCs, mean ± SD = 4.57 ± 3.15 vs. 3.56 ± 1.43; *p* = NS) (Figure 4b). N-D ALS patients showed a 67% of IgG1 positivity (AUC = 0.61), while IgG1 levels of L-S ALS patients showed a significant difference compared to HCs (ALS vs. HCs, mean ± SD = 4.82 ± 3.02 vs. 3.56 ± 1.43; *p* = 0.017) (Figure 4c). L-S ALS patients showed 81% of positivity to IgG1 (AUC = 0.68), while no difference was observed between IGg1 levels in N-D ALS patients with respect to L-S. No difference was shown between IgG4 levels in the total ALS group (N-D and L-S) compared to HCs (ALS vs. HCs, mean ± SD = 0.54 ± 0.42 vs. 0.45 ± 0.32; *p* = NS) (Figure 4d). In a similar way, IgG4 levels did not show a significant difference between N-D ALS and HCs (N-D ALS vs. HCs, mean ± SD = 0.35 ± 0.38 vs. 0.45 ± 0.32; *p* = NS) (Figure 4e), while IgG4 levels of L-S ALS *p* were significantly different compared to HCs (L-S ALS vs. HCs, mean ± SD = 0.66 ± 0.46 vs. 0.45 ± 0.32; *p* = 0.011) (Figure 4f). Indeed, L-S ALS patients showed 81% positivity to IgG4 in serum (AUC = 0.69). No difference was observed between IgG4 levels in N-D ALS patients compared to L-S.

### 3.9. Correlation Analyses

**Figure 5:**
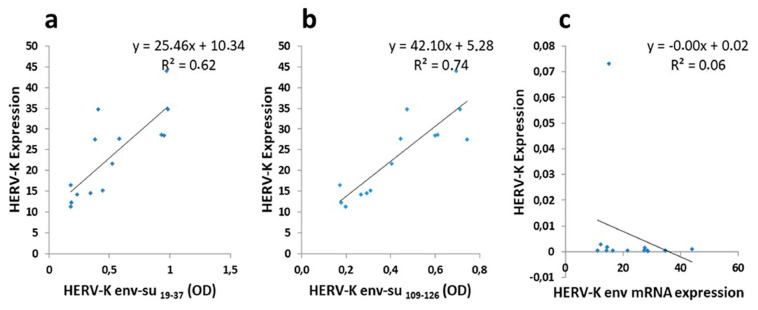
Correlation analysis. Scatter plot showing the correlations between (**a**,**b**) IgG against HERV-K env _19–37_ and HERV-K env _109–126_ in serum compared to HERV-K env expression on the B cells membrane of ALS patients and between (**c**) HERV-K env expression on B cells membrane compared to the HERV-K transcripts in PBMCs.

A correlation analysis was performed to establish the association between the antibody positivity toward the selected peptides in serum and the HERV-K-env expression on the B cells membrane of ALS patients. We found a positive correlation regarding HERV-K env su _19–37_ antibodies in serum compared to HERV-K-env expression on the B cells membrane (R^2^ = 0.6207; *p* = 0.0008) (Figure 5a). A very good correlation was also observed for HERV-K env su _109–126_ peptide antibodies in serum compared to HERV-K-env expression on the B cells membrane (R^2^ = 0.7449; *p* < 0.0001) (Figure 5b). Instead, no correlation was found between HERV-K env expression on B cells membrane compared to the HERV-K transcripts in PBMCs (Figure 5c).

## 4. Discussion

ALS is characterized by heterogeneous clinical signs and symptoms, and a definite diagnosis may require 13 to 18 months, once of other disorders that can affect upper and lower motor neurons have been excluded [11,12].

The identification of specific and sensitive circulating biomarkers for ALS could permit an early diagnosis and avoid unnecessary and potentially harmful therapeutic trials. In particular, an increase in HERVs expression has been observed in some neurological diseases, including ALS, although there is no evidence that this may be the primary causative factor for the pathology.

In a previous study, we documented an HERV-K env antigen humoral immunity response by detecting specific IgG antibodies in serum and CSF of Sardinian patients affected by ALS, with respect to other neurological diseases [8]. In this study, to better secure the role of retroviruses in ALS, and validate the use of HERV-K as a possible diagnostic biomarker, we expanded the size of our previously studied cohort to 55, and respective healthy controls to 102, confirming the data obtained in the previous paper [8]. We also used different methodological approaches to better understand the role of HERV-K on humoral and cell-mediated immune responses both by using cytometric analysis to quantify the HERV-K env peptides expression on the PBMCs membrane of ALS patients and controls and by investigating HERV-K transcripts in PBMCs by RT-qPCR method. Notably, PBMCs have been proposed as a good non-invasive option for studying ALS [13].

The analysis of viral protein expression on lymphomonocytes showed that HERV-K env protein was expressed with greater percentage frequency in B cells of ALS patients. This finding supports the results of ELISA tests in serum and CSF showing that the production of specific immunoglobulins towards selective retroviral peptides was significantly higher in patients with ALS with respect to HCs [8]. Instead, the analysis of the HERV-K env transcripts has not proven any significant difference between patients and controls as previously observed by Garson [14]. Interestingly, other HERVs families have been associated to different neurological diseases, such as HERV-W to multiple sclerosis (MS) from different authors [15,16], and HERV-W env expression on the membrane of PBMCs in MS patients seems modulated by natalizumab [17]. Recently, we have also shown that circulating antibody levels directed against the HERV-W env-su _93–108_ and HERV-W env-su _248–262_ peptide fragments are different in different CNS demyelinating disorders [18,19].

Based on our findings, HERV-K env protein appeared to be poorly expressed on the CD4+ and CD8+ lymphocyte membranes of both ALS patients and controls (data not shown), despite retroviral antigens also determining a humoral response T helps mediate.

The specific immune response which T and B mediated after stimulation of PBMCs with two antigenic peptides derived from the HERV-K env-su glycoprotein, namely HERV-K env _19–37_ and HERV-K env _109–126_, in patients with ALS and HCs, showed significant differences among the cytokines produced, indicating a good and broad reactivity of the immune system. In particular, the expression of IL-6 by CD19/B cells was significantly different after stimulation with HERV-K env _109–126_ peptide in ALS patients in comparison to HCs, suggesting that this peptide is likely responsible for the B-cell activation, considered the autocrine activity of this cytokine. Instead, the HERV-K env _19–37_ peptide seems able to foster a pro-inflammatory response by stimulation of CD19/B cells and a statistically significant greater production of IFN-γ. In CD19/B cells, both peptides were not able to stimulate the TNF-α production, while the HERV-K env _19–37_ peptide induced a greater production of TNF-α in CD8/T cells.

The mechanism for this latter effect is unknown. We believe it might be due to an insufficient immune innate response in ALS patients after a 6-hour stimulus of PBMCs. This is consistent with an increased retroviral expression observed on monocytes of HCs compared to patients, as TNF-α is a pro-inflammatory cytokine that appears at the early stages of the innate immune response. Previous studies have reported higher levels of IL-6 in ALS patients in comparison to HCs [20,21,22,23,24] and rising IL-6 levels in plasma were also associated with risk for disease progression [20]. Interestingly, although IL-6 may have an established bivalent role in inflammation, namely both a pro and anti-inflammatory action, a peripheral IL-6 upregulation usually is related to an inflammatory cell response also able to induce an endothelial cells damage at blood–brain barrier. [24]. Thus, our data support the possibility that an increased expression of HERV-K can stimulate IL-6 and IFN-γ determining a chronic inflammatory state through a T lymphocytes/Th1-type cytokines response which, in patients genetically predisposed, may play a role in worsening the disease course.

Moreover, we studied the expression of MIP-1α and MCP-1 by CD19/B-cells stimulated with both peptides, and found a significant increase in the expression of MIP-1α in ALS patients. This finding fits previous data in the literature showing either a negative correlation between expression of MIP-1α and disease progression rate or a positive correlation with disease duration, thus suggesting a possible protective role of this chemokine on ALS outcome [25]. The antigenic specific stimulation generated by HERV-K env _19–37_ and HERV-K env _109–126_ contribute to the specific increase in MIP-1 α levels as observed in serum and CSF by other authors [21,25]. Regarding MCP-1, we document a lower expression by CD19/B cells in ALS patients compared to HCs, following stimulation with both peptides. As higher MCP-1 levels were associated with worse disease severity and faster progression by several authors [21], and due to the HERV-K related lower expression of this chemokine documented in our study, it is likely to work together with the increased expression of MIP-1α to produce a better ALS outcome.

In this context, in order to investigate which IgG subclass of antibodies anti-HERV-K were predominant in patients with ALS, we determined plasma levels of IgG1 and IgG4 immunoglobulins. This is an important issue in order to clarify if the high levels of IgG found in ALS are protective (IgG4) or harmful (IgG1) [26]. Indeed, recent data have indicated that IgG4 antibodies may fulfil a protective role dampening the more harmful effects of IgG1 when directed against the same epitopes [26]. In addition, we evaluated if the observed IgG1 and IgG4 production could be correlated with disease progression and prognosis. We detected IgG1 plasma levels significantly higher in ALS patients compared to HCs; conversely, no difference between the two groups was observed for IgG4 plasma levels. Of note, IgG1 levels were not significantly different in newly diagnosed ALS patients with respect to long-survivor patients, thus this humoral finding does not have prognostic significance.

Interestingly, in long-survivor ALS patients, IgG1 and IgG4 plasma levels were both significantly increased with respect to HCs. This data indicates that, in long-survivors, the detrimental effect of increased IgG1 levels in plasma may be progressively dampened by the parallel increment in plasma of IgG4 levels. The mechanisms behind the pathological contribution of HERV-K activation in ALS remain yet to be completely clarified. Here, we document divergent mechanisms, related to humoral and cell-mediated immune response to antigenic peptides derived from HERV-K in ALS patients. In this context, our study provides useful information to better understand the possible role of endogenous retroviruses in neurodegenerative diseases. In particular, we highlighted the ability of HERV-K to modulate the immune system at different levels, generating mediators mainly involved in proinflammatory response. Of note, despite depletion, B cells of ALS patients express higher levels of HERV-K env protein compared to HCs feeding the inflammatory loop, by induction of a continuous proinflammatory stimulus of T lymphocytes sustained by exposition of viral protein on B lymphocytes membrane. Furthermore, we better understand the role of the cytokine IL 6 in the early stage of ALS as responsible for the injury of the BBB, such that to support neuronal damage. Certainly, an early knowledge is sorely needed to diagnose ALS, as recently described by Keon et al. [27]. Adequate translation of our results into clinical practice may speed up the use of HERV-K as biomarker of disease progression due to its ability to modulate cytokines and chemokines as mediators of inflammation partly responsible for motor neurons damage.

## Figures and Tables

**Table 1:** Demographic data of investigated groups and methodological approaches used.

	Groups, *n*°	Age, Years, Mean ± SD	Sex, Males/Females, *n*°	Kind of Analysis
ALS HCs	14 11	62.2 ± 8.4 63 ± 9.3	9/5 9/2	Flow Cytometry
ALS HCs	36 44	65.3 ± 9.3 61.3 ± 10.8	24/12 27/17	Real-time PCR
ALS HCs	55 102	65 ± 9.8 63.4 ± 11.	34/21 60/42	ELISA
ALS HCs	48 58	65 ± 9.6 63 ± 10.6	17/19 34/24	Immunonephelometry

**Table 2:** Primers and probes for quantitative real-time PCR.

Target Gene	Primer Sequence 5′-3′
HERV-K env Fw	5′-TTGTCTGTTGTTAGTCTGC-3′
HERV-K env Rev	5′-CAATCTGATCTCTCTTGCTTT-3′
HERV-K env TaqMan^®^Probe	FAM-5′-TCCGAAGAGACAGCGACCATC-3′-TAMRA
GAPDH Fw	5′-CAAGGAGTAAGACCCCTGGAC-3′
GAPDH Rw	5′-TCTACATGGCAACTGTGAGGAG-3′
GAPDH TaqMan^®^Probe	ROX-5′-ACCAGCCCCAGCAAGAGCACAAGA-3′-BQ2

**Table 3:** Distribution of peripheral blood cells subset in HCs and patients with Amyotrophic Lateral Sclerosis.

Cell Type	HCs (*n* = 11)	ALS (*n* = 14)	*p* Value
CD3-	52.66 ± 10.02%	22.11 ± 5.11%	0.01502
CD19	44.76 ± 11.17%	17.311 ± 7.48%	0.03434
CD3	53.59 ± 13.25%	68.54 ± 8.97%	0.02002
CD4	40.00 ± 12.57%	52.41 ± 13.49%	ns
CD8	13.59 ± 4.48%	16.13 ± 7.38%	ns
CD14	5.3 ± 2.25	3.6 ± 1.97%	ns
CD56/CD16	2.3 ± 0.73	1.9 ± 0.62%	ns

## Data Availability

The datasets used and/or analyzed during this study are available on reasonable request from the corresponding author.

## Abbreviations

ALS = Amyotrophic Lateral Sclerosis; HERV-K = human endogenous retrovirus of the K family; and CSF = Cerebrospinal Fluid.
